# Highly Trained Female Runners Show Greater Durability and Physiological Resilience Than Performance‐Matched Male Counterparts

**DOI:** 10.1111/sms.70299

**Published:** 2026-05-09

**Authors:** Diego Jaén‐Carrillo, Christina D. Bruce, Justin S. Lawley, Michele Zanini

**Affiliations:** ^1^ Department of Sport Science University of Innsbruck Innsbruck Austria; ^2^ School of Education, Childhood, Youth and Sport, the Open University Milton Keynes UK; ^3^ School of Sport, Exercise and Health Sciences, Loughborough University Loughborough UK

**Keywords:** fatigue, marathon, performance, running economy, sex differences, trail running, uphill running

## Abstract

Endurance performance determinants are typically assessed under fresh conditions, although physiological and mechanical characteristics deteriorate during prolonged exercise, a phenomenon recently termed durability or resilience. However, it is unclear whether durability and mechanisms underpinning it differ between sexes. Eleven females and 11 males matched for performance completed three laboratory visits including: a graded exercise test, a 12‐min uphill time trial (TT), and a 180‐min treadmill run at standardized moderate intensity, interspersed with repeated TTs every 60 min (total distance: 36 ± 3 and 42 ± 3 km, respectively). Physiological, biomechanical, and neuromuscular variables were assessed throughout the steady‐state run and TTs and analyzed with linear mixed models. Time‐trial performance declined during the run, with females displaying smaller speed decrements than males after 3 h (−1 vs. −10%, *p* < 0.01) and smaller reductions in carbohydrate oxidation and respiratory exchange ratio during steady‐state (*p* < 0.05) and TTs (*p* < 0.01). Males displayed larger reductions in peak blood lactate during TTs (*p* < 0.001), while peak heart rate (HR) and oxygen uptake remained unchanged in both groups. During the prolonged run, females showed greater resilience for maximal isometric knee extensor force, HR, and perceived exertion (*p* < 0.05), whereas running economy deteriorated similarly between sexes. Biomechanical adjustments occurred in both sexes, with larger alterations observed in males for TTs (stride length; *p* < 0.05) and in females during steady‐state (contact time and stiffness; *p* < 0.05). In conclusion, highly trained female runners demonstrate greater durability than performance‐matched males. Sex differences are characterized by superior metabolic and neuromuscular resilience in females, whereas biomechanical changes appear similar between sexes. Finally, whether these findings persist under distance‐matched conditions warrants investigation.

## 1 Introduction

Endurance running performance can be primarily explained by maximal oxygen uptake (V̇O_2_max), the fractional utilization of V̇O_2_max at lactate threshold, and running economy (RE) [1]. Although these determinants are typically assessed under fresh conditions, recent evidence has shown that they progressively deteriorate during prolonged exercise [2, 3, 4, 5, 6]. Building on this, the capacity of an athlete to attenuate fatigue‐induced declines in physiological parameters has been conceptualized as durability or physiological resilience [7, 8], where durability refers to performance‐related outcomes, and resilience to the physiological changes underpinning it [9].

During prolonged running, durability and resilience have been primarily assessed through physiological parameters, with studies reporting drifts in RE [4, 5], heart rate (HR) [10], and V̇O_2_max [5], subsequently eliciting a nonlinear decline in speed at lactate threshold (LT) [5]. These shifts seem to be linked to the progressive depletion of muscle glycogen stores, which necessitates the compensatory recruitment of less efficient Type‐II muscle fibers to maintain a given speed [11]. Biomechanically, during continuous running the body often adopts a “smoother” running style in response to fatigue, presumably to minimize the repetitive stress of impact forces [12, 13]. This protective transition is typically characterized by an increased cadence [14] and ground contact time [15], as well as reduced stride length [14], and a higher duty factor [15]. While these mechanical adjustments aim to mitigate tissue damage and pain, they also seem to contribute to an upward drift in RE [12, 16]. Biomechanical responses during prolonged running also seem to differ between sexes, with elite male marathoners experiencing larger decreases in stride length between late‐race laps than females, as well as cadence decreases attributed to increased contact time [17].

Although evidence of sex‐differences in durability is lacking, females seem to demonstrate superior neuromuscular fatigue resistance (i.e., resilience) compared to males [18, 19, 20, 21, 22, 23], with differences in knee extension and flexion [24] and smaller perturbations in strength‐related capabilities, even when performance‐matched to males [2, 25]. Metabolically, females may demonstrate greater resilience because of a higher proportion of type‐I fibers [26, 27] and a superior fat oxidation, which can spare glycogen during prolonged submaximal efforts [28, 29]. Sex‐differences in metabolic changes have been shown to be consistent with neuromuscular fatigue associated with running, with females displaying little to no changes in RE and maximal knee strength following a 2 h run at the first ventilatory threshold, whilst declining in male runners [24]. Conversely, evidence from ultra‐trail efforts exceeding 100 km found that RE deteriorated at a similar rate for both sexes, suggesting that the female neuromuscular advantage may not always grant a proportional edge in RE resilience [13, 18]. Notably, the specific mechanical demands of trail terrain may amplify performance differences between sexes. During uphill exercise, the performance gap typically widens to 18%–22% (compared with 10%–12% on level terrain), primarily linked to differences in lean‐to‐fat mass ratios [30]. Integrating these sex‐specific factors is therefore useful for accurately isolating the mechanisms underlying durability and for optimizing performance strategies across variable gradients during running. Considering the above background, evidence of sex‐differences in durability and resilience are currently limited and unclear, and an evaluation of metabolic, neuromuscular, and biomechanical parameters under well‐controlled conditions may provide a mechanistic insight for possible differences in fatigued performance between sexes.

The present study aimed to examine sex‐dependent differences in durability and physiological, biomechanical, and neuromuscular resilience during 3 h of running in highly‐trained trail runners. Specifically, we investigated (i) sex differences in physiological responses and running gait during a steady‐state prolonged run at moderate intensity, and (ii) sex differences in performance, neuromuscular function, and mechanical responses during repeated uphill time‐trials performed in an unfatigued state and throughout the prolonged run. Exercise duration was standardized rather than distance, as this approach allows the progressive cardiovascular, metabolic, and neuromuscular drifts that underpin durability [4, 5, 10, 11, 24] to be tracked at equivalent time points across all participants, irrespective of their absolute running speed. We hypothesized that (i) physiological and biomechanical variables would worsen during the prolonged run, with females better able to maintain unfatigued parameters, and (ii) that these differences would be reflected in the performance testing, with females having smaller reductions in time‐trial speed and lesser deterioration of metabolic and biomechanical responses.

## 2 Methods

### 2.1 Participants

Eleven male and eleven female highly‐trained trail runners (Tier 3 and 4 based on a recent sport performance framework [31]; Table 1) volunteered to participate in the study. Written informed consent was provided, and the study was approved by the Ethics Committee of the University of Innsbruck (no. 109/2024). Inclusion criteria required participants to be ≥ 18 years, have ≥ 2 years of trail running experience, a weekly running volume ≥ 50 km with ≥ 800 m of positive elevation gain, and to be free from musculoskeletal injury at the time of testing. Male and female participants were performance‐matched based on the International Trail Running Association (ITRA) performance index level (Table 1). The participants' ITRA performance index and age were selected as the matching criteria to normalize competitive performance for sex, age, and race distance, thereby positioning each athlete relative to their own sex‐specific normative distribution [32]. This approach ensures that both groups occupy an equivalent competitive tier within their respective populations, which is the methodologically appropriate benchmark when the research question concerns intrinsic sex differences in physiological responses rather than differences in absolute aerobic capacity [33]. Sample size was determined based on previous research examining sex differences in prolonged running [24] reporting large interaction effects for RE and knee extensor strength. For effects within this range, at least 7 participants per group were estimated to achieve 80% statistical power at *α* = 0.05. Accordingly, the present sample provides ~95% power to detect interaction effects of similar magnitude.

**TABLE 1:** Participants and trial characteristics.

	Males	Females	*p*
Participants			
Sample size (*n*)	11	11	—
Age (y)	29 ± 6	29 ± 4	0.96
Height (m)	1.80 ± 0.06	1.66 ± 0.06	< 0.001
Body mass (kg)	72.9 ± 8.7	60.9 ± 6.2	0.001
BMI	20.2 ± 1.5	18.4 ± 1.6	0.02
Training experience (y)	6 ± 2	7 ± 4	0.47
Training volume (km/wk)	88 ± 24	66 ± 23	0.04
Training elevation (m/wk)	3309 ± 919	2436 ± 997	0.05
ST frequency (sessions/wk)	1.5 ± 0.8	1.4 ± 0.7	0.58
ITRA points	703 ± 88	579 ± 64	0.001
ITRA level	Advanced 1	Advanced 1	—
Baseline trials			
Speed at LT (km/h)	13.7 ± 0.9	11.5 ± 0.7	0.001
GXT max speed (km/h)	18.6 ± 1.5	16.1 ± 1.3	0.001
Time trial speed (km/h)	10.3 ± 0.5	8.2 ± 0.7	< 0.001
Time trial peak V̇O_2_ (mL/kg/min)	55.5 ± 3.5	44.7 ± 4.2	< 0.001
Time trial peak HR (beats/min)	184 ± 8	182 ± 6	0.81
iMVC (N)	635 ± 150	434 ± 73	< 0.001
iMVC (N/kg)	8.7 ± 2.0	7.2 ± 1.2	0.04
Prolonged run trial			
Speed (km/h)	12.1 ± 1.2	10.2 ± 0.8	< 0.001
Pre‐post body mass change (kg)	−2.1 ± 0.9	−1.0 ± 0.4	< 0.001
Pre‐post body mass change (%)	−2.7 ± 1.3	−1.7 ± 0.8	< 0.001
Steady‐state EE (Kcal)	2376 ± 303	1665 ± 151	< 0.001
Time trials EE (Kcal)	697 ± 92	482 ± 44	< 0.001
Total EE (Kcal)	3073 ± 38	2147 ± 187	< 0.001
Total EE (Kcal/kg)	41.4 ± 4.2	34.6 ± 3.8	< 0.001
Total distance covered (km)	42.2 ± 3.4	35.5 ± 2.6	< 0.001

Abbreviations: BMI, body mass index; EE, energy expenditure; GXT, graded exercise test; HR, heart rate; iMVC, maximal isometric voluntary contraction; ITRA, international trail running association; LT, lactate threshold; ST, strength training; V̇O_2_, oxygen uptake.

### 2.2 Experimental Overview

Participants attended the laboratory on 3 occasions within a 14‐day period, and they were instructed to refrain from strenuous exercise, caffeine, and alcohol consumption for 24 h prior to each visit. They were also asked to record their diet and physical activity in the 48 h preceding visit 1 and replicate them in visit 2 and 3. Additionally, participants were required to wear the same running shoes across all visits. Participants completed 3 laboratory visits: an incremental graded exercise test (GXT) to determine lactate threshold (LT; visit 1), a 12 min uphill time‐trial performed in an unfatigued state (visit 2), and a 180 min treadmill run at submaximal intensity, interspersed with a TT performed every 60 min. Physiological and biomechanical measures were repeatedly assessed during the prolonged run and TTs while neuromuscular measures were assessed at the end of every hour during the prolonged run, before and immediately after each TT. Repeated uphill TTs were incorporated to operationalize durability as the change in maximal performance capacity at multiple time points throughout the prolonged run [7, 9]. All tests were performed on a motorized treadmill (HP Cosmos Pulsar 4P, HP Cosmos Sports & Medical, Germany) under standardized environmental conditions (21°C–24°C, 45%–55% relative humidity), with an airflow of 2.55 m/s provided by a fan positioned 1.5 m in front of the participant, and heart rate (HR) was continuously monitored (H10, Polar, Finland).

### 2.3 Visit 1: Graded Exercise Test

In visit 1, participants' height and body mass (BM) were measured upon arrival with a calibrated scale (MPB 300 K100, Kern & Sohn, Germany) and stadiometer (213, SECA GmbH, Germany), respectively. Participants were then familiarized with performing maximal isometric voluntary contractions (iMVC) of the knee extensors while seated on a custom‐built dynamometer. Voluntary force was recorded using a PowerLab data acquisition system and LabChart 8 software (ADInstruments, New Zealand). Thereafter, LT was assessed through a GXT. After completing a 5 min warm‐up at a speed corresponding to a rating of perceived exertion (RPE; 6–20 scale) of 6–7, they performed a treadmill incremental test consisting of 6–8 stages of 3 min, at speed increments of 1 km/h per stage, until volitional exhaustion was reached, consistent with previous incremental testing protocols in runners [5, 34, 35]. A 20 μL capillary blood sample (E‐T‐E Capillaires, Hirschmann GmbH, Germany) was collected from the earlobe at the end of each stage and analyzed for blood lactate concentration (B[La^−^]; SUPER GL ambulance, Dr. Müller Gerätbau GmbH, Germany). LT was defined as the highest velocity preceding a systematic rise in B[La^−^] (~0.5 mmol/L above resting values) [34, 36]. To standardize intensity across participants in the moderate exercise domain, the speed of the prolonged run was set at 85% of the speed corresponding to LT + 0.5 mmol/L.

### 2.4 Visit 2: Unfatigued Time Trial

Seventy‐two hours after the GXT, participants visited the laboratory to perform a TT in an unfatigued state. Once BM was measured and participants performed a standardized warm‐up, they completed a 12‐min uphill time trial at a + 12% gradient (TT) [37] in a fresh condition aiming to maximize distance covered. Treadmill speed was self‐selected and adjusted manually by an investigator in response to participants' hand signals. Throughout the test, participants wore a low‐dead‐space mask to measure gas composition of inspired and expired air via an open‐circuit metabolic cart (ML206, ADInstruments, NSW, Australia) with a heated pneumotach amplifier (Series 1110, Hans Rudolph Inc., USA) and gas mixing chamber (MLA246, ADInstruments). The inspired and expired gas volumes and concentrations were continuously sampled, with these analyzers calibrated before each test using a known gas mixture (16% O_2_ and 5% CO_2_) and ambient air. The turbine volume transducer was calibrated using a 3‐L syringe (Hans Rudolph, KS). The volume and concentration signals were time aligned, accounting for the transit delay in capillary gas and analyzer rise time relative to the volume signal. Heart rate (HR), B[La^−^], RPE, and iMVC were recorded before and immediately after the TT. Maximal HR (HR_max_) was also recorded at the end of the TT.

### 2.5 Visit 3: Prolonged Run and Fatigued Time Trials

**FIGURE 1:**
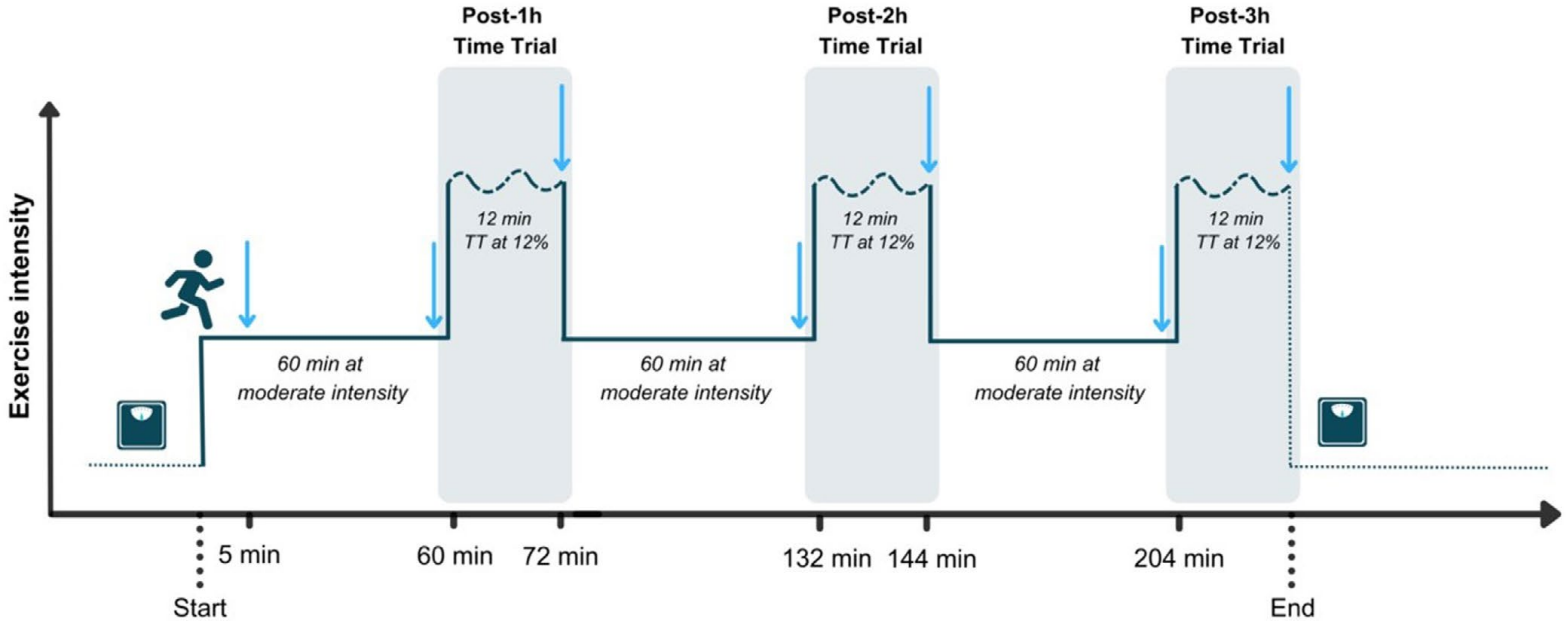
Graphical representation of the prolonged run protocol. The thin line indicates the exercise intensity during each stage of the protocol. The weighting scale icon indicates when body mass was measured, and arrows indicate time points for the collection and analysis of physiological, biomechanical, neuromuscular, and perceptual parameters. See methods for extensive details.

In the third visit, participants' BM, resting B[La^−^], and unfatigued iMVC were measured upon arrival. Then, a 180 min steady‐state treadmill run was completed at moderate intensity standardized as described above. Every 60 min, uphill TTs were performed to assess fatigued performance (i.e., post‐1 h TT, post‐2 h TT, post‐3 h TT; Figure 1). Respiratory gases were measured intermittently (in 5 min bins) during the prolonged run at 5, 60, 120, and 180 min. However, respiratory gases were measured continuously during each TT. Biomechanics‐related data were sampled for 1 min at the same time points through a foot‐mounted inertial sensor (Stryd Next Gen, Stryd Inc., Boulder, CO, USA). RPE was assessed at the same time points and at the end of each TT. B[La^−^] and iMVC were assessed before and after each TT, and BM was measured at the end of the run to assess sweat rate. During the prolonged run, a carbohydrate (CHO) gel solution (Crown Sport Nutrition, Spain) was provided at a rate of 90 g/h, and water was consumed ad libitum, with fluid intake measured upon consumption.

### 2.6 Metabolic Measurements

Oxygen uptake (V̇O_2_) and carbon dioxide consumption (V̇CO_2_) values were averaged over 3‐min for metabolic‐related measures during the prolonged run, while the highest 30 s average was used for measures during the TTs. V̇O_2_ and V̇CO_2_ were used to calculate respiratory exchange ratio (RER), running economy (RE) expressed in mL/kg/km, fat and carbohydrate oxidation rates (Fat_ox_ and CHO_ox_, respectively), and energy expenditure (EE). To account for the effect of BM changes on RE, BM pre‐ to postprolonged run was measured and linear regression was used to estimate BM at each measurement time point, which was then used to calculate RE. Nonprotein respiratory quotient equations were used to estimate CHO_ox_ and Fat_ox_ (g/min). The energy derived from each substrate was calculated by multiplying fat and CHO utilization by 9.75 and 4.07 Kcal, respectively [38]. Energy expenditure was calculated as the sum of the energy derived from fat and CHO.

### 2.7 Biomechanical Measurements

The following spatiotemporal metrics were calculated from a foot‐mounted inertial sensor: cadence (steps per minute), stride length normalized to leg length (0.53 x height) [39, 40], ground contact time, duty factor, vertical oscillation, and leg stiffness. Duty factor was calculated as the ratio between ground contact time and total stride time (ground contact time + flight time). The validity and reliability of the sensor‐derived metrics have been previously reported [41, 42].

### 2.8 Neuromuscular Measurements

Maximal isometric voluntary contraction (iMVC) of the knee extensors was assessed immediately before and after the control and fatigued TTs to quantify neuromuscular fatigue. Participants were seated on a custom‐built dynamometer chair with the hip and knee at ~90° of flexion. The lower leg was attached to a rigid lever arm connected to a calibrated load cell (model 9UB, HBM, Germany) and recorded via LabChart 8 (ADInstruments, New Zealand); the load cell signal was zeroed prior to each testing session to account for signal offset. Prior to the main protocol, participants performed two submaximal contractions (~50% of perceived maximum) separated by 30 s of rest as familiarization and neuromuscular activation. Two to three 3‐s maximal contractions, where force output was within 5% of at least two, were performed before the protocol began, separated by 90 s of rest. The highest torque value was retained as the unfatigued iMVC for analysis. Single iMVCs were completed within 45 s of each time trial to minimize interference with subsequent running performance [43, 44]. Real‐time visual feedback of force output and strong standardized verbal encouragement were provided throughout [45].

### 2.9 Statistical Analysis

Baseline differences between sexes were assessed using independent‐samples *t*‐tests. Separate linear mixed models (LMMs) were fitted for each dependent variable, with Sex (female, male), Time (steady‐state: 5, 60, 120, and 180 min; time trials: CON, post‐1 h, post‐2 h, post‐3 h), and their interaction (Sex × Time) specified as fixed effects with participant ID included as a random intercept. To assess the potential confounding effect of the distance covered or energy expenditure (EE) during the run on the dependent variables, each was added as a covariate for separate LMMs analysis. Model assumptions were verified by inspection of residual normality and homoscedasticity. When significant Sex × Time interactions were detected, contrast analyses were conducted to examine sex differences in absolute changes between time points, with Bonferroni adjustments for multiple comparisons. Changes are reported as percentage differences relative to baseline. Effect sizes are reported as partial eta squared (*η* ^2^ₚ) and interpreted as small (0.01–0.06), medium (0.06–0.14), and large (> 0.14). The threshold for significance was fixed at *p* ≤ 0.05.

## 3 Results

### 3.1 Baseline and Trials Characteristics

Participants and trial characteristics are reported in Table 1, with data analyzed for the 11 performance‐matched pairs of males and females. Sex‐specific performance status did not differ between males (703 points) and females (579 points), based on ITRA points and level (“Advanced 1” level points: 700–725 for males, 575–599 for females; Table 1). Groups did not differ in training experience or strength training frequency (*p* ≥ 0.66), but males had a higher weekly training volume and elevation than females (*p* = 0.03; Table 1). Males had higher speed at LT and during the unfatigued 12‐min TT (*p* < 0.001). During the prolonged run, the cumulative energy expenditure differed between sexes, with females expending less energy relative to body mass (Kcal/kg) than males during the steady‐state part of the run and across the 12‐min TT (−16%; *p* < 0.001). Similarly, the total distance covered by females was 16% lower than that of males (*p* < 0.001; Table 1).

### 3.2 Steady‐State Responses During the Prolonged Run

**FIGURE 2:**
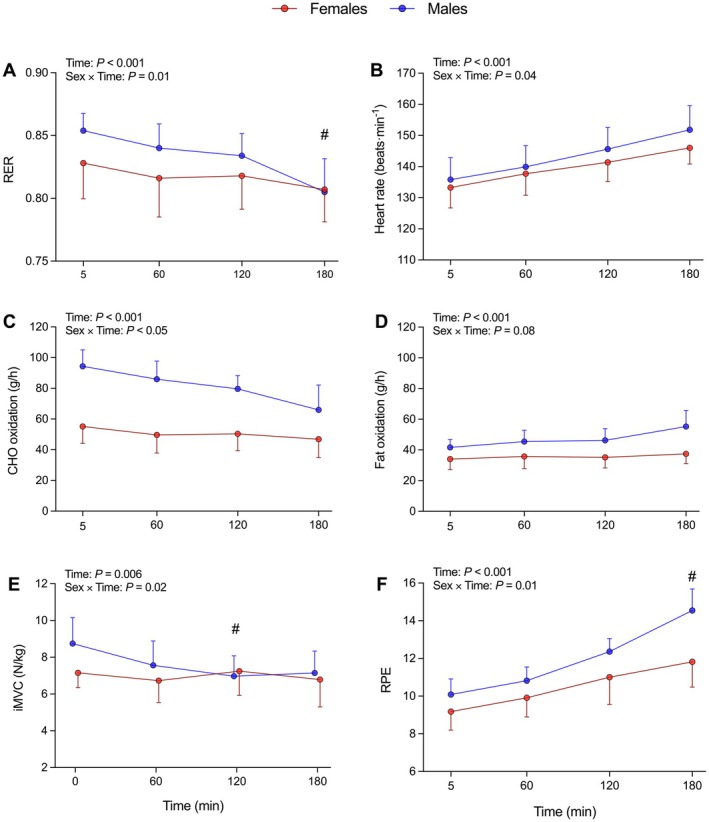
Physiological and neuromuscular responses during the 3 h steady‐state run at moderate intensity. Data are expressed as mean ±95% confidence intervals (females = 11, males = 11). Between‐sex difference in change from unfatigued at that time point: #*p* ≤ 0.05.

Most physiological, perceptual, and mechanical responses changed during the prolonged run. A main effect of Time was observed for CHO_ox_, Fat_ox_, and RER (*p* < 0.001; *η* ^2^ₚ ≥ 0.28) with a shift towards fat utilization during the trial, and a Sex × Time interaction for CHO_ox_ and RER (*p* < 0.05; *η* ^2^ₚ ≥ 0.12) indicating a larger change in males compared to females (at 3 h: CHO_ox_: −29% vs. −9%, Figure 2C), with contrast analysis revealing different RER changes after 3 h (−5.6 vs. −2.1%; *p* < 0.05; Figure 2A). A main effect of Sex was found for CHO_ox_, Fat_ox_ (*p* ≤ 0.01; *η* ^2^ₚ ≥ 0.28; Figure 2C,D) with higher utilization of both fuel sources in males, although there was no main effect of Sex on RER (*p* = 0.32).

HR, RPE, and relative iMVC followed a common pattern, with a main effect of Time (*p* ≤ 0.003; *η* ^2^ₚ ≥ 0.20) exhibiting an upward drift in HR and RPE, with a decrease in iMVC, and a Sex × Time interaction (*p* ≤ 0.04; *η* ^2^ₚ ≥ 0.12; Figure 2B,E,F, respectively). Specifically, at 3 h females showed smaller changes than males for RPE (+30% vs. +47%; *p* = 0.03; Figure 2F), while relative iMVC differed between sexes at 2 h (−18% [males] vs. 0% [females]; *p* = 0.02; Figure 2E). A main effect of Time was found for RE and B[La^−^] (*p* ≤ 0.03), both increasing as exercise progressed, although no main effects of Sex or Sex × Time interaction were found (*p* ≥ 0.14; Table 2).

**TABLE 2:** Changes during 3 h of running at steady state.

	Group	% change from 5 min			Sex effect			Time effect			Sex × Time effect		
		1 h	2 h	3 h	*F*	*p*	*η* ^2^ₚ	*F*	*p*	*η* ^2^ₚ	*F*	*p*	*η* ^2^ₚ
Running economy (mL/kg/km)	Females	1.1 ± 3.4	1.7 ± 3.4	2.8 ± 4.2	0.00	0.93	0.00	3.33	0.03	0.14	1.57	0.24	0.06
	Males	−0.3 ± 3.2	−0.6 ± 5.7	1.4 ± 5.2									
Blood lactate (mmol/L)	Females	—	21.8 ± 18.2	34.3 ± 26.7	2.06	0.17	0.09	5.79	0.01	0.22	2.08	0.14	0.09
	Males	—	3.9 ± 34.2	20.9 ± 56.4									
Duty factor (%)	Females	1.0 ± 2.6	2.3 ± 2.8	2.6 ± 3.2	1.07	0.31	0.05	0.75	0.52	0.03	1.92	0.13	0.09
	Males	0.4 ± 4.1	−0.1 ± 3.0	−0.6 ± 4.1									
Vertical oscillation (cm)	Females	1.8 ± 7.0	−0.8 ± 5.8	−1.8 ± 8.0	0.77	0.39	0.03	1.40	0.25	0.06	0.88	0.45	0.04
	Males	3.6 ± 6.4	2.3 ± 6.7	3.0 ± 8.7									

**FIGURE 3:**
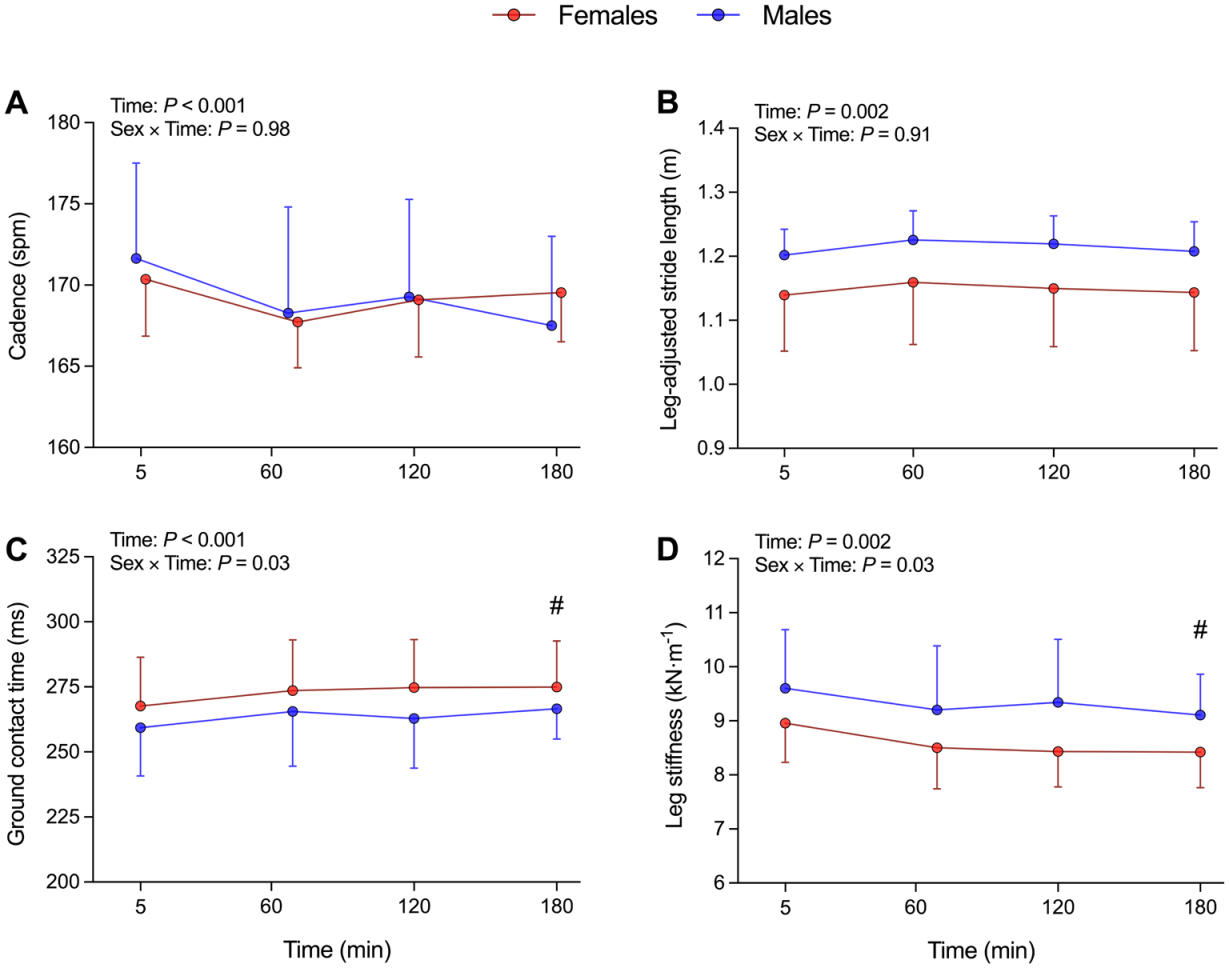
Biomechanical responses during the 3 h steady‐state run at moderate intensity. Data are expressed as mean ±95% confidence intervals (females = 11, males = 11). Between‐sex difference in change from unfatigued at that time point: #*p* ≤ 0.05.

When running mechanics variables were analyzed, cadence, ground contact time, stiffness, and stride length changed similarly, with a main effect of Time (*p* < 0.01; *η* ^2^ₚ ≥ 0.21; Figure 3B) showing decreased stiffness and stride length, and increased ground contact time and cadence. No Time effects were found for duty factor and vertical oscillation (*p* ≥ 0.13; *η* ^2^ₚ ≤ 0.08; Table 2). A Sex × Time interaction was only found for contact time and leg stiffness (*p* ≤ 0.03; *η* ^2^ₚ ≥ 0.13; Figure 3), with females showing a larger increase in contact time (+2.8 vs. +0.7%; *p* = 0.03; Figure 3C), and a greater decline in leg stiffness (−5.8 vs. −1.5%; *p* = 0.04; Figure 3D). No main effects of Sex were found for any running mechanics metric (*p* ≥ 0.11; *η* ^2^ₚ ≤ 0.11; Figure 3; Table 2).

When total distance or EE (in Kcal/kg) was included as a covariate to separate LMMs, effects remained unchanged for Time and Sex × Time interactions, suggesting that the shorter distance covered by females had limited confounding effects on the changes during the prolonged run. Details can be found in the supplemental materials (Table S1).

### 3.3 Time Trial Responses

**FIGURE 4:**
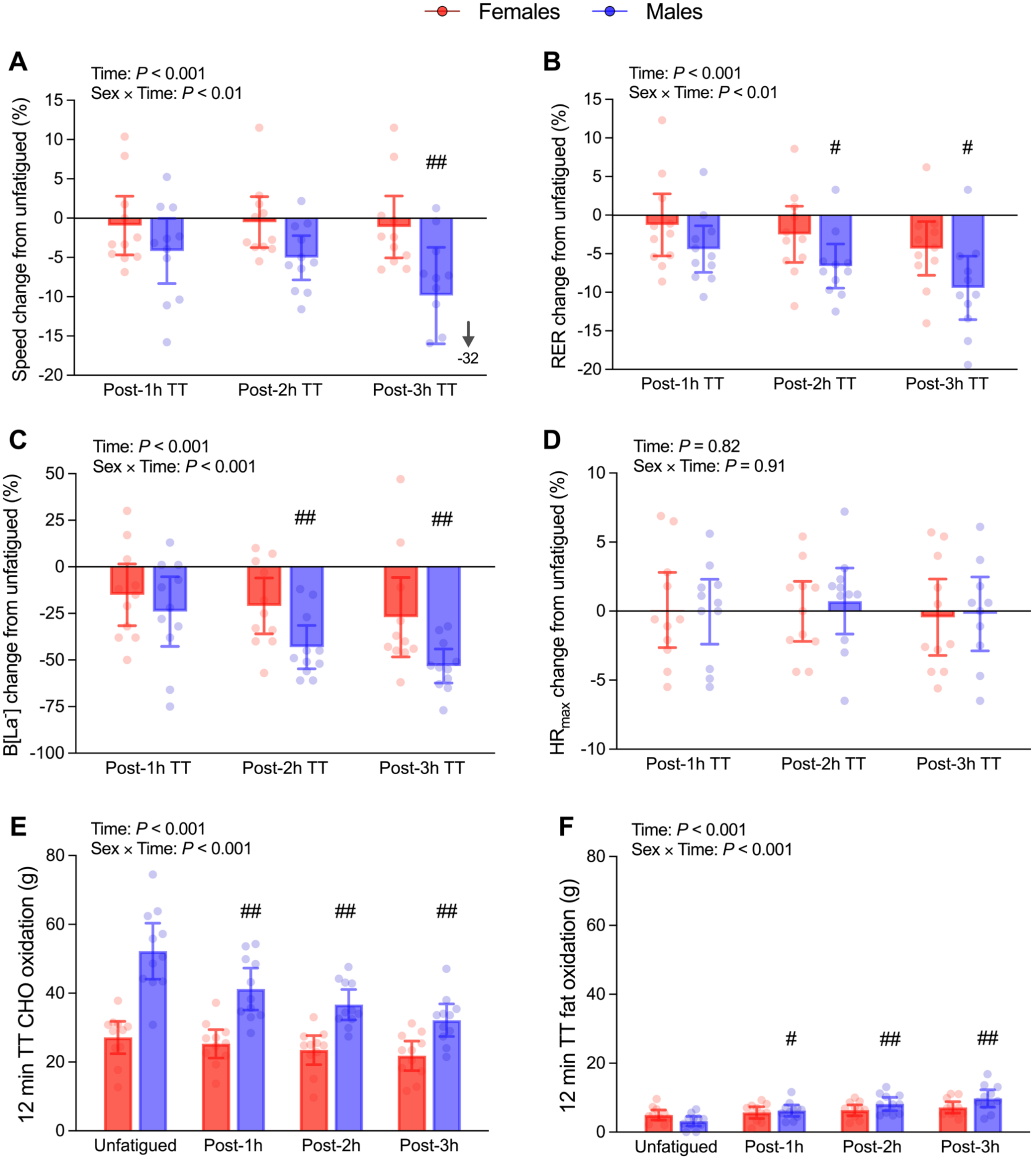
Performance and physiological changes in 12 min uphill time trials (TT) compared to unfatigued during 3 h steady‐state run at moderate intensity. Panels A–D show percentage change from the unfatigued TT; panels E–F show absolute values for each condition. Data are expressed as mean ±95% confidence intervals (females = 11, males = 11), with dots representing individual responses. Between sex difference in change from unfatigued at that time point: #*p* ≤ 0.05; ##*p* ≤ 0.01.

Compared to an unfatigued state, TT performance declined as the prolonged run progressed, with a main effect of Time and Sex observed for TT speed (*p* < 0.001; *η* ^2^ₚ ≥ 0.29). TT speed also demonstrated a Sex × Time interaction (*p* = 0.003; *η* ^2^ₚ = 0.20), with females displaying smaller decreases in the post‐3 h TT (−1.1% vs. −9.9%; *p* = 0.003; Figure 4A).

Metabolic responses followed a similar pattern, with a main effect of Time for peak B[La^−^], RER, CHO_ox_, and Fat_ox_ (*p* < 0.001; *η* ^2^ₚ ≥ 0.63; Figure 4B,C, Figure 4E,F, respectively), showing a shift from CHO to fat oxidation and decreased peak B[La^−^], and a Sex effect for peak B[La^−^] and CHO_ox_ (*p* ≤ 0.049; *η* ^2^ₚ ≥ 0.16; Figure 4E,F, Figure 4C). Sex × Time interactions were found for all these variables (*p* < 0.009; *η* ^2^ₚ ≥ 0.17; Figure 4B,C, Figure 4E,F), with females showing smaller metabolic perturbations. For peak B[La^−^], males displayed a larger decrease than females from unfatigued (7.44 vs. 5.54 mmol/L) to post‐2 h TT (−43 vs. −21%; *p* = 0.003) and post‐3 h TT (−53 vs. −27%; *p* = 0.006), reaching 3.38 vs. 3.82 mmol/L, respectively (Figure 4C). Males were also found to have a larger decrease in RER from unfatigued (0.96 vs. 0.91) to post‐2 h TT (−6.6 vs. −2.5%; *p* = 0.03) and post‐3 h TT (−9.0 vs. −4.3%; *p* = 0.02), both reaching 0.87 at the end of the trial (Figure 4B). Males showed a greater reduction in CHO_ox_ during the post‐2 h TT (−28 vs. −8%; *p* < 0.001) and post‐3 h TT (−36 vs. −16%; *p* < 0.001), alongside a greater increase in Fat_ox_ at the same time points (156 vs. 45% and 214 vs. 61%, respectively; *p* < 0.001; Figure 4E,F).

Other physiological variables showed more heterogeneous responses. No main or interaction effects were found for HR (*p* ≥ 0.54; *η* ^2^ₚ ≤ 0.02; Figure 4D) which remained unchanged throughout the TTs, whilst RPE increased with time (Time effect: *p* ≤ 0.001), although no Sex or Sex × Time effects were found (*p* ≥ 0.07; Table 3). Peak V̇O_2_ showed an effect of Sex (*p* < 0.001), with males having a higher peak V̇O_2_, but no Time or Sex × Time effects were found (*p* ≥ 0.79; Table 3), indicating that both groups maintained their peak V̇O_2_ during the TT even at the end of the prolonged run.

**TABLE 3:** Changes between 12 min uphill time trials (TT).

	Group	% change from baseline TT			Sex effect			Time effect			Sex × Time effect		
		Post‐1 h TT	Post‐2 h TT	Post‐3 h TT	*F*	*p*	*η* ^2^ₚ	*F*	*p*	*η* ^2^ₚ	*F*	*p*	*η* ^2^ₚ
Peak V̇O_2_ (mL/min/kg)	Females	−0.4 ± 1.9	0.1 ± 7.4	0.8 ± 6.6	51.08	< 0.001	0.70	0.34	0.79	0.02	0.30	0.83	0.00
	Males	−0.5 ± 2.0	−2.8 ± 8.5	0.3 ± 6.7									
RPE	Females	−2.8 ± 7.3	2.7 ± 5.6	5.9 ± 6.5	3.58	0.07	0.14	22.97	< 0.001	0.53	2.37	0.08	0.10
	Males	−4.6 ± 7.2	−2.2 ± 6.8	1.5 ± 3.5									
Cadence (steps/min)	Females	−1.0 ± 2.9	−0.5 ± 2.3	−0.3 ± 2.1	0.99	0.33	0.04	2.10	0.11	0.09	0.06	0.98	0.00
	Males	−1.3 ± 2.5	−0.8 ± 3.0	−1.2 ± 3.7									
Ground contact time (ms)	Females	3.3 ± 4.5	3.2 ± 5.6	3.8 ± 6.2	1.42	0.25	0.06	12.15	< 0.001	0.36	1.30	0.28	0.06
	Males	5.5 ± 6.3	6.0 ± 5.1	9.9 ± 8.8									

Abbreviations: RPE, rating of perceived exertion; V̇O_2_, oxygen uptake.

**FIGURE 5:**
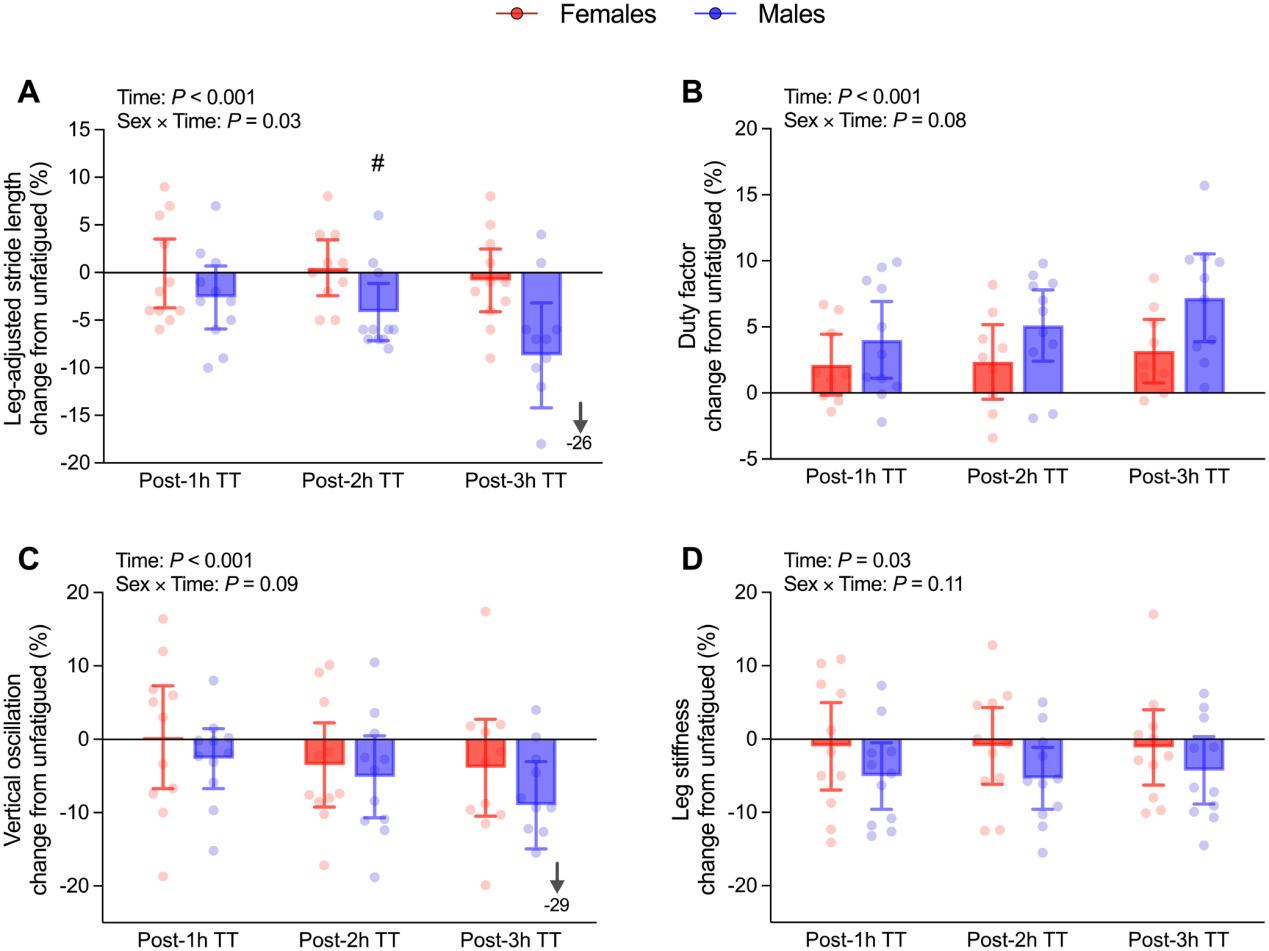
Biomechanical changes in 12 min uphill time trials (TT) compared to unfatigued during 3 h steady‐state run at moderate intensity. Data are expressed as mean ±95% confidence intervals (females = 11, males = 11), with dots representing individual responses. Between sex difference in change from unfatigued at that time point: #*p* ≤ 0.05.

Among running mechanics metrics, a main effect of time was found for ground contact time, duty factor (Figure 5B), vertical oscillation, stiffness (Figure 5D), and stride length (Figure 5A) (*p* ≤ 0.03; *η* ^2^ₚ ≥ 0.12) but not for cadence (*p* = 0.11; *η* ^2^ₚ = 0.09), with CT and duty factor increasing, whilst vertical oscillation, stiffness, and stride length decreased (Figure 5; Table 3). No Sex or Sex × Time effects were found for any metric (*p* ≥ 0.11; *η* ^2^ₚ ≤ 0.09; Figure 5; Table 3) except for stride length. Leg‐adjusted stride length showed a Sex × Time interaction (*p* = 0.03; *η* ^2^ₚ = 0.12), with larger changes post‐2 h TT in males (−0.8 vs. −8.7%; *p* = 0.04; Figure 5A).

Similar to the steady‐state results, when total distance or EE (in Kcal/kg) was included as a covariate to separate LMMs, effects remained unchanged for Time and Sex × Time interactions. Details can be found in the supplemental materials (Table S2).

## 4 Discussion

The present study examined sex‐differences in performance durability and physiological, biomechanical, and neuromuscular resilience during a 3 h run interspersed by uphill time trials (TT) every 60 min, in highly‐trained trail runners matched for performance level. We found that females exhibit superior durability compared to males, with a smaller decline in uphill TT speed following the prolonged run (−1% vs. −10%). The durability difference was underpinned by greater physiological resilience, characterized by a smaller shift from CHO_ox_ to Fat_ox_ and smaller cardiovascular drift during steady‐state running. Similar results were found during TTs, with smaller reductions in B[La^−^], CHO_ox_, and RER in females from 2 h onwards. Furthermore, females showed preservation of knee extensor force throughout the prolonged run, while males experienced a substantial decline from 2 h. Interestingly, while both sexes showed deterioration in RE and biomechanical parameters such as increased ground contact time and reduced leg stiffness, changes were not sex‐dependent. Collectively, these results suggest that female runners have better durability than males, although the higher distance covered by males during the trial may partially explain the results. Sex‐differences in durability seem to be mediated by metabolic and knee extension resilience, rather than divergent mechanical alterations.

### 4.1 Uphill Performance Durability

This study demonstrated that females exhibit superior durability during uphill running compared to males, with a smaller reduction in 12 min uphill TT performance after 3 h of running (−1% vs. −10%). Although sex differences in durability have not been reported in runners, our findings align with a recent laboratory‐based study in cycling [46], whereas they differ from field data in professional cyclists, where males have been found to have better durability than females when matched for total accumulated work [47] or work above critical power [48]. These contrasting results may depend on the exercise matching prescription—duration in our work and in Pastorio et al. [46] vs. work in previous research—as highlighted above, as well as differences in racing patterns between males and female professional cyclists. That females maintained TT performance and metabolic stability after 3 h, even when total distance was included as a covariate to the statistical models, suggests that the observed durability advantage reflects a genuinely more resilient physiological profile under time‐equivalent stress. This interpretation is further supported by the fact that sex differences in metabolic and neuromuscular outcomes were substantial from 2 h onwards, when the absolute dose discrepancy between groups, while present, was smaller than at the end of the trial. Admittedly, the difference in distance covered (−16% in females) could have overestimated sex differences at standardized distances, although the effect of standardizing exercise by duration or distance has been shown to elicit similar durability results [4]. It would therefore be interesting for future investigations to assess the effects of distance vs. duration matching when assessing sex differences in durability.

Sex differences in duration‐matched durability seem to be underpinned by physiological factors rather than biomechanical. Specifically, during TTs, differences were found for B[La^−^], RER, and CHO_ox_, substantially reduced in males and showing greater metabolic perturbations than females, while similar changes were found for HR, RPE, and peak V̇O_2_, demonstrating similar levels of effort during TTs throughout the 3 h run. Neuromuscular fatigue could also explain some of the differences, with a higher reduction of iMVC in males from 2 h onwards. On the other hand, mechanical parameters showed similar alterations across sexes during TT. In males, the reduction in speed was accompanied by a shortening of stride length and a prolongation of ground contact time, indicating that the decline in performance was mechanically underpinned by alterations in spatiotemporal parameters rather than by speed reduction alone. These findings provide practical evidence that females have higher durability than males during prolonged running. The preservation of iMVC in females observed here is consistent with earlier evidence of lower neuromuscular fatigability in females during isometric and single‐limb dynamic tasks [49, 50]. However, sex differences in neuromuscular fatigability during whole‐body dynamic exercise are more equivocal [51, 52], with a recent study [46] using a duration‐matched design similar to ours finding no sex differences in voluntary force, voluntary activation, or contractile function following 90 min of heavy‐intensity cycling, suggesting that metabolic and oxidative factors may be the primary drivers of the female durability advantage. As highlighted above, these duration‐matched findings warrant confirmation when distance or work are standardized between sexes.

### 4.2 Physiological and Neuromuscular Outcomes: Steady State Run

During the steady‐state run, females displayed smaller drifts in physiological parameters (HR, RER, substrate oxidation, RPE, and iMVC), while RE and B[La^−^] increased similarly between sexes. Similarly, in the fatigued TT males demonstrated greater reductions in peak B[La^−^], RER, and CHO_ox_, suggesting that females have a better physiological resilience than males when matched for exercise duration. These findings are consistent with previous research indicating that females rely more on Fat_ox_ than males during prolonged exercise, particularly during the first ~120 min of activity [29, 53]. In line with this, the RER has frequently been reported to be lower in females during prolonged, low‐intensity exercise, supporting a lower reliance on whole‐body CHO_ox_ [53]. For example, lower RER values have been observed in females (0.87) compared with males (0.94) during a submaximal prolonged run [29] and higher maximal rates of Fat_ox_ in females have been reported during an incremental test in a large cohort of athletes [54]. Given the high reliability of physiological responses during prolonged running recently demonstrated [55], it is unlikely that these results are confounded by excessive within‐participant variability. Neuromuscular fatigue outcomes are also in line with previous research [24, 56], reporting declines of 18% and 36% in knee extension strength in males following long‐distance running performed in laboratory and field settings, respectively. These studies conducted their protocols either on a flat treadmill [24] or on the trails incorporating downhill sections [56], whereas our study did not include any downhill running, thus minimizing the likelihood of observing temporary muscle damage.

Taken together, these findings indicate that although physiological parameters drift differently between sexes during duration‐matched exercise (e.g., HR and RER), these may not reflect in RE or B[La^−^] differences at submaximal intensity, while TT efforts exacerbate these alterations due to the maximal effort associated with the task (e.g., speed, peak B[La^−^], and substrate oxidation). This may suggest that, during prolonged running, athletes of different characteristics may not display large disparities at submaximal intensity, while TTs may be more sensitive as a measure of durability and resilience. Mechanistically, the higher physiological resilience of females could be mediated by their higher proportional area of fatigue‐resistant Type‐I muscle fibers [26, 27] and greater muscle capillarization compared to males [28, 57]. Such physiological traits may allow for enhanced Fat_ox_ and subsequent glycogen sparing, delaying the metabolic perturbations that can also trigger premature strength loss in males [28, 29, 33]. The lower resilience in males is also supported by research showing that females experience lesser deoxygenation of the knee extensors during high‐intensity efforts, permitting them to sustain higher relative intensities for longer [13, 49]. As highlighted above, these findings are based on an exercise duration‐matching design and warrants confirmation when distance or work are standardized between sexes.

### 4.3 Biomechanical Outcomes

Biomechanically, both sexes adopted a “smoother” gait strategy to minimize impact forces, characterized by increased cadence and ground contact time, alongside reduced stride length and leg stiffness, a phenomenon well‐documented in graded running where step frequency increases and swing phases shorten [37]. The larger deteriorations in stride length seen in males post‐3 h TT might also relate to their greater iMVC loss (i.e., neuromuscular fatigue) [18] which could constrain the ability to sustain uphill speed. This is in line with studies generally reporting an increased ground contact time and reduced iMVC during fatigued running, although their correlation is not consistently supported [58, 59]. Similarly, repeated 30 min bouts of downhill running induced an increase in ground contact time and duty factor alongside iMVC loss, with changes more pronounced in the initial exercise bout, although no correlations were found between changes in mechanics metrics and iMVC [60]. Importantly, these sex‐specific differences did not translate into a greater deterioration in RE during the steady‐state run. Taken together, these findings suggest that uphill TTs may represent a sensitive task during which neuromuscular fatigue can be associated with some spatiotemporal gait alterations, allowing for detection of subtle durability alterations during prolonged exercise.

### 4.4 Limitations

This study is not without limitations. Firstly, although males and females were pair‐matched by ITRA performance index levels, running speeds were higher in males, resulting in a greater distance covered (42.2 km vs. 35.5 km) and a 16% difference in total EE (Kcal/kg). This difference may partially explain the more pronounced declines in their TT performance and metabolic stability compared to the females, although including total distance covered or EE as covariate in the statistical models elicited similar outcomes. As a time‐invariant covariate cannot fully account for progressive differences in dose accumulation, distance‐matched experiments would be needed to expand these duration‐matched findings. Secondly, while treadmill running allows for high internal validity and standardized environmental conditions, it does not fully replicate the complexity of outdoor running, especially the irregular trail surfaces or the technical demands of varying outdoor gradients. Additionally, it should be noted that quantifying posterior chain muscle groups (e.g., gastrocnemii) alongside knee extensor strength would have provided a more comprehensive assessment of neuromuscular fatigue associated with running. Third, a fixed +12% gradient was used for TT assessment, and durability may manifest differently on level running, inclines of different gradients, or on downhill segments. Furthermore, the validity of Stryd‐derived biomechanical variables is not uniformly established under the conditions of this study. Ground contact time and, therefore, duty factor, is systematically underestimated relative to optical reference systems [42, 61]. Similarly, leg stiffness derived from the spring‐mass model relies on assumptions of symmetric ground contact mechanics that may be less applicable during steep uphill running, where contact geometry differs from level locomotion [62]. As specific validation data for this sensor under inclined treadmill conditions are not yet available in the literature, findings related to leg stiffness from the uphill time trials should be interpreted cautiously. Additionally, males ran at a higher absolute speed than females (12.1 vs. 10.2 km/h), which may have independently contributed to the greater biomechanical and neuromuscular perturbations observed, as higher running velocities impose greater mechanical loading per stride regardless of relative intensity. This potential confound cannot be fully dissociated from intrinsic sex differences in resilience within the present design.

### 4.5 Conclusions

In conclusion, highly trained female trail runners demonstrated superior durability compared to performance‐matched males during a 3 h run at moderate intensity, with smaller decrements in repeated 12 min uphill TT performance and greater resilience of metabolic and neuromuscular parameters, while biomechanical adaptations were broadly similar between sexes, with sex‐specific differences limited to contact time and leg stiffness during the steady‐state run, and stride length during the time trials. This durability advantage appears to be mediated by a higher resilience of metabolic and neuromuscular systems, with smaller reductions in CHO_ox_, iMVC, and peak B[La^−^] in females during steady state and TT efforts, although differences in distance covered (−16% in females) may have partially emphasized these differences. These findings reinforce the value of durability and resilience [8, 9] in distinguishing athletes with similar performance characteristics in a fresh state. From a practical perspective, the data suggest that training and racing strategies should be sex‐specific, with male athletes likely requiring greater focus on interventions that delay the onset of fatigue, particularly when repeated high‐intensity efforts are involved, and a more conservative pacing strategy in races.

### 4.6 Perspective

The durability advantage hereby observed in females (preserved uphill TT performance, smaller metabolic perturbation, and maintained knee extensor force) indicates that sex‐specific physiological resilience modulates the effective performance ceiling during prolonged efforts in ways that unfatigued profiling cannot predict. For race pacing, male runners should adopt a more conservative pacing strategy (especially uphill) during long‐duration events, restrain effort on early ascents, and build in longer recovery windows between high‐intensity segments. Female runners, by contrast, appear to sustain a higher effective intensity ceiling for longer, suggesting that a more even split strategy may be physiologically supported. Importantly, some of the differences found may be smaller at matched distances (rather than durations), with potential implications for applied practice.

In this study, sex differences in physiological resilience were larger and more consistent during TTs than during the steady‐state phase. This has implications for durability monitoring in training, in which brief maximal efforts interspersed within prolonged training runs, structured similarly to the TT protocol used here, may provide a more sensitive measure of individual durability than submaximal physiological monitoring alone. Practitioners and coaches can use this approach to identify athletes whose performance degrades disproportionately under cumulative fatigue. Future studies could expand these findings to level running by including repeated high‐intensity efforts during marathon‐like runs as a durability assessment.

## Funding

The authors have nothing to report.

## Conflicts of Interest

The authors declare no conflicts of interest.

## Supporting information


**Table S1:** Statistical outcomes of changes during 3 h of running at steady state, with the inclusion of “total distance covered” as a covariate for the linear mixed models. Contrast analysis reveals changes between 5 min (fresh) and following 1 h, 2 h, and 3 h.
**Table S2:** Statistical outcomes of changes between 12 min uphill time trials, with the inclusion of “total distance covered” as a covariate for the linear mixed models. Contrast analysis reveals changes between unfatigued and post‐1 h, post‐2 h, and post‐3 h.

## Data Availability

The data that support the findings of this study are available from the corresponding author upon reasonable request.
