# Supplementary material for: Highly Trained Female Runners Show Greater Durability and Physiological Resilience Than Performance‐Matched Male Counterparts
Source: Scand J Med Sci Sports. 2026 May 9;36:e70299. doi: 10.1111/sms.70299 (PMC13156839; doi:10.1111/sms.70299)
Supplement: Supplementary file 1 — Table S1: Statistical outcomes of changes during 3 h of running at steady state, with the inclusion of “total distance covered” as a covariate for the linear mixed models. Contrast analysis reveals changes between 5 min (fresh) and following 1 h, 2 h, and 3 h. Table S2: Statistical outcomes of changes between 12 min uphill time trials, with the inclusion of “total distance covered” as a covariate for the linear mixed models. Contrast analysis reveals changes between unfatigued and post‐1 h, post‐2 h, and post‐3 h. [file SMS-36-e70299-s001.docx]

**Supplementary Materials**

**Highly trained female runners show greater durability and physiological resilience than performance-matched male counterparts**

**Authors:** Diego Jaén-Carrillo ^1^, Christina D. Bruce ^1^, Justin S. Lawley ^1^, Michele Zanini ^2,3^

^1^ Department of Sport Science, University of Innsbruck, Innsbruck, Austria

^2^ School of Education, Childhood, Youth and Sport, The Open University, Milton Keynes, UK

^3^ School of Sport, Exercise and Health Sciences, Loughborough University, Loughborough, UK

* Correspondence to:

Dr. Michele Zanini (ORCID: 0009-0007-8148-8843)

School of Education, Childhood, Youth and Sport

The Open University, Milton Keynes, MK7 6AA, United Kingdom

E-mail: [michele.zanini@open.ac.uk](mailto:michele.zanini@open.ac.uk)

**Table S1.** Statistical outcomes of changes during 3 h of running at steady state, with the inclusion of “total distance covered” as a covariate for the linear mixed models. Contrast analysis reveals changes between 5 min (fresh) and following 1 h , 2 h, and 3 h.

|  | **Sex effect** | | | **Time effect** | | | **Sex × Time effect** | | | **Sex × Time contrast analysis** | | |
| --- | --- | --- | --- | --- | --- | --- | --- | --- | --- | --- | --- | --- |
|  | **F** | **P** | **η²ₚ** | **F** | **P** | **η²ₚ** | **F** | **P** | **η²ₚ** | **1 h** | **2 h** | **3 h** |
| Running Economy | 0.14 | 0.710 | 0.00 | 3.33 | 0.025 | 0.14 | 1.57 | 0.240 | 0.06 | - | - | - |
| RER | 3.45 | 0.080 | 0.15 | 13.35 | <0.001 | 0.42 | 4.08 | 0.011 | 0.18 | 1.000 | 1.000 | 0.050 |
| CHO oxidation | 12.63 | 0.002 | 0.39 | 9.49 | <0.001 | 0.32 | 2.67 | 0.049 | 0.12 | 1.000 | 0.707 | 0.120 |
| Fat oxidation | 0.24 | 0.630 | 0.01 | 7.73 | <0.001 | 0.28 | 2.34 | 0.080 | 0.11 | - | - | - |
| Blood Lactate | 0.35 | 0.560 | 0.02 | 3.26 | 0.049 | 0.14 | 3.11 | 0.055 | 0.13 | N/A | - | - |
| Heart Rate | 0.27 | 0.610 | 0.01 | 96.36 | <0.001 | 0.82 | 2.88 | 0.040 | 0.12 | 1.000 | 1.000 | 0.180 |
| RPE | 0.19 | 0.660 | 0.00 | 71.08 | <0.001 | 0.77 | 4.06 | 0.010 | 0.16 | 1.000 | 1.000 | 0.030 |
| iMVC/kg | 0.26 | 0.620 | 0.01 | 5.26 | 0.003 | 0.20 | 3.38 | 0.020 | 0.14 | 1.000 | 0.022 | 0.820 |
| Cadence | 1.24 | 0.280 | 0.05 | 6.19 | <0.001 | 0.23 | 0.06 | 0.980 | 0.00 | - | - | - |
| Contact Time | 14.82 | <0.001 | 0.40 | 12.31 | <0.001 | 0.37 | 3.16 | 0.030 | 0.13 | 1.000 | 0.370 | 0.033 |
| Duty Factor | 14.01 | 0.001 | 0.39 | 0.74 | 0.530 | 0.03 | 1.92 | 0.130 | 0.09 | - | - | - |
| Vertical Oscillation | 1.72 | 0.200 | 0.07 | 1.38 | 0.260 | 0.06 | 0.92 | 0.430 | 0.04 | - | - | - |
| Stiffness | 1.86 | 0.190 | 0.08 | 10.29 | <0.001 | 0.33 | 3.28 | 0.030 | 0.14 | 0.950 | 0.180 | 0.040 |
| Adjusted Stride Length | 5.96 | 0.020 | 0.21 | 4.78 | 0.004 | 0.19 | 0.37 | 0.780 | 0.02 | - | - | - |

**Table S2.** Statistical outcomes of changes between 12 min uphill time trials, with the inclusion of “total distance covered” as a covariate for the linear mixed models. Contrast analysis reveals changes between unfatigued and post-1h , post-2h, and post-3h.

|  | **Sex effect** | | | **Time effect** | | | **Sex × Time effect** | | | **Sex × Time contrast analysis** | | |
| --- | --- | --- | --- | --- | --- | --- | --- | --- | --- | --- | --- | --- |
|  | **F** | **P** | **η²ₚ** | **F** | **P** | **η²ₚ** | **F** | **P** | **η²ₚ** | **Post-1h** | **Post-2h** | **Post-2h** |
| Speed | 14.88 | <0.001 | 0.40 | 8.71 | <0.001 | 0.29 | 5.18 | 0.003 | 0.19 | 0.373 | 0.070 | 0.003 |
| Blood Lactate | 1.04 | 0.320 | 0.05 | 45.96 | <0.001 | 0.68 | 6.96 | <0.001 | 0.24 | 1.000 | 0.004 | 0.006 |
| RER | 8.82 | 0.007 | 0.28 | 40.21 | <0.001 | 0.66 | 4.24 | 0.009 | 0.17 | 0.210 | 0.030 | 0.020 |
| CHO oxidation | 19.91 | <0.001 | 0.47 | 35.91 | <0.001 | 0.63 | 12.43 | <0.001 | 0.37 | 0.005 | <0.001 | <0.001 |
| Fat oxidation | 2.53 | 0.130 | 0.10 | 36.97 | <0.001 | 0.63 | 11.19 | <0.001 | 0.34 | 0.018 | <0.001 | <0.001 |
| Heart Rate | 1.49 | 0.240 | 0.06 | 0.32 | 0.810 | 0.01 | 0.18 | 0.910 | 0.00 | - | - | - |
| RPE | 1.10 | 0.310 | 0.05 | 22.94 | <0.001 | 0.05 | 2.39 | 0.077 | 0.10 | - | - | - |
| Peak Oxygen Uptake | 9.69 | 0.005 | 0.31 | 0.97 | 0.410 | 0.04 | 1.09 | 0.360 | 0.05 | - | - | - |
| Contact Time | 1.28 | 0.270 | 0.06 | 12.10 | <0.001 | 0.04 | 1.28 | 0.290 | 0.06 | - | - | - |
| Duty Factor | 2.12 | 0.160 | 0.09 | 17.44 | <0.001 | 0.45 | 2.37 | 0.080 | 0.10 | - | - | - |
| Vertical Oscillation | 2.41 | 0.130 | 0.10 | 8.06 | <0.001 | 0.27 | 2.24 | 0.090 | 0.09 | - | - | - |
| Stiffness | 0.97 | 0.330 | 0.04 | 3.05 | 0.030 | 0.12 | 2.09 | 0.110 | 0.09 | - | - | - |
| Adjusted Stride Length | 1.16 | 0.290 | 0.05 | 6.24 | <0.001 | 0.22 | 3.09 | 0.033 | 0.12 | 1.000 | 0.044 | 0.300 |
| Cadence | 0.00 | 0.970 | 0.00 | 2.11 | 0.110 | 0.09 | 0.06 | 0.980 | 0.00 | - | - | - |
